# Role of piRNA biogenesis and its neuronal function in the development of neurodegenerative diseases

**DOI:** 10.3389/fnagi.2023.1157818

**Published:** 2023-05-03

**Authors:** Kaoru Sato, Ken-ichi Takayama, Satoshi Inoue

**Affiliations:** ^1^Department of Systems Aging Science and Medicine, Tokyo Metropolitan Institute for Geriatrics and Gerontology (TMIG), Tokyo, Japan; ^2^Integrated Research Initiative for Living Well with Dementia (IRIDE), Tokyo Metropolitan Institute for Geriatrics and Gerontology (TMIG), Tokyo, Japan

**Keywords:** piRNA, PIWI, neurodegenerative disease, aging, biomarker, dementia, neuron

## Abstract

Neurodegenerative diseases, such as Alzheimer’s disease (AD), Parkinson’s disease (PD), and amyotrophic lateral sclerosis (ALS), are caused by neuronal loss and dysfunction. Despite remarkable improvements in our understanding of these pathogeneses, serious worldwide problems with significant public health burdens are remained. Therefore, new efficient diagnostic and therapeutic strategies are urgently required. PIWI-interacting RNAs (piRNAs) are a major class of small non-coding RNAs that silence gene expression through transcriptional and post-transcriptional processes. Recent studies have demonstrated that piRNAs, originally found in the germ line, are also produced in non-gonadal somatic cells, including neurons, and further revealed the emerging roles of piRNAs, including their roles in neurodevelopment, aging, and neurodegenerative diseases. In this review, we aimed to summarize the current knowledge regarding the piRNA roles in the pathophysiology of neurodegenerative diseases. In this context, we first reviewed on recent updates on neuronal piRNA functions, including biogenesis, axon regeneration, behavior, and memory formation, in humans and mice. We also discuss the aberrant expression and dysregulation of neuronal piRNAs in neurodegenerative diseases, such as AD, PD, and ALS. Moreover, we review pioneering preclinical studies on piRNAs as biomarkers and therapeutic targets. Elucidation of the mechanisms underlying piRNA biogenesis and their functions in the brain would provide new perspectives for the clinical diagnosis and treatment of AD and various neurodegenerative diseases.

## 1. Introduction

Over the past two decades, advances in next-generation sequencing (NGS) technologies have led to the discovery of numerous small non-coding RNAs (sncRNAs), including small interfering RNAs (siRNAs), microRNAs (miRNAs), and P-element-induced wimpy testis (PIWI)-interacting RNAs (piRNAs) (Siomi et al., 2011; Czech et al., 2018; Sato and Siomi, 2020; Wu and Zamore, 2021; Wang X. et al., 2022). These small RNAs engage in a gene silencing mechanism called RNA silencing, in which gene expression is negatively regulated by sequence-specific sncRNAs (Kim et al., 2009; Siomi et al., 2011).

**Figure 1:**
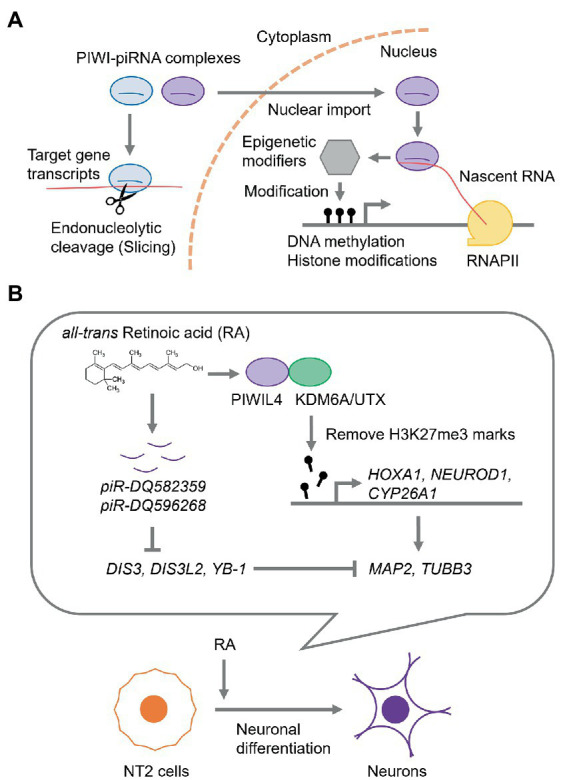
piRNA-mediated regulation of gene expression. **(A)** The current model for piRNA-mediated RNA silencing mechanism. In the cytoplasm, piRNA guides PIWI protein to the complementary target RNA to catalyze the endonucleolytic cleavage (slicing). While, in the nucleus, PIWI-piRNA complexes recognize nascent RNAs and recruit epigenetic modifiers, such as DNA methyltransferases, histone modifiers, and chromatin mark readers, thereby modifying the chromatin state and gene expression. RNAPII, RNA polymerase II. **(B)** A gene regulation *via* piRNAs during neuronal differentiation of NT2 cells.

In piRNA-mediated RNA silencing, piRNAs form a complex with PIWI proteins, which are highly conserved Argonaute protein family members, and guide the PIWI-piRNA complex to gene transcripts with complementary sequences to silence their expression at the transcriptional and post-transcriptional levels (Kim et al., 2009; Siomi et al., 2011; Sato and Siomi, 2018; Figure 1A). Humans possess four *PIWI* genes: *HIWI/PIWIL1*, *HILI/PIWIL2*, *PIWIL3*, and *HIWI2/PIWIL4* (Sasaki et al., 2003). Notably, piRNAs exhibit features distinct from those of miRNAs; e.g., miRNAs have 18–24 nucleotides (nt), whereas piRNAs are 26–31 nt in length (Vagin et al., 2006; Brennecke et al., 2007). In addition, piRNAs are the most diverse class of sncRNAs; e.g., in the human genome, over 27 million of unique piRNA sequences are deposited in a piRNA database, piRBase, whereas only about 1,900 miRNAs are encoded in the genome according to the miRNA database miRbase (Zhang et al., 2014; Kozomara et al., 2019; Wang J. et al., 2022). Most piRNAs are processed from transcripts encoded by specific genome loci, called piRNA clusters, which are rich in transposon remnants (Brennecke et al., 2007; Goriaux et al., 2014; Parhad and Theurkauf, 2019). Additionally, transcripts of protein-coding genes and long non-coding RNA (lncRNAs) can also be the source of piRNAs (Aravin et al., 2006; Girard et al., 2006; Siomi et al., 2011; Ku and Lin, 2014; Wang and Lin, 2021).

In almost all animals, piRNAs are abundantly expressed in the gonadal cells, such as the testes and ovaries, where they silence transposons to maintain germline genome stability (Kim et al., 2009; Siomi et al., 2011). Strikingly, an increasing number of studies have demonstrated that the expression of piRNAs is not limited to the germline but occurs more broadly in other non-gonadal somatic cells, including neuronal cells (Rojas-Ríos and Simonelig, 2018; Kim, 2019; Perera et al., 2019; Wakisaka et al., 2019). In the human brain, numerous piRNAs are produced and play important roles in various neuronal processes, including neuronal differentiation, and in the development of various neurodegenerative diseases (Kim, 2019; Perera et al., 2019; Wakisaka et al., 2019; Huang and Wong, 2021; Lauretti et al., 2021). This review focuses on the neuronal expression and functions of piRNAs, mainly in humans, the implication of dysregulation of neuronal piRNAs in various human neurodegenerative diseases, and piRNAs as potential biomarkers and therapeutic targets against these diseases.

## 2. Neuronal piRNA functions

In this section, we summarize multiple neuronal functions of piRNAs and their biogenesis factors.

### 2.1. Neuronal expression of piRNAs

Expression of piRNAs in the brain has been detected in animal species (Ghildiyal et al., 2008; Dharap et al., 2011; Lee et al., 2011; Rajasethupathy et al., 2012; Qiu et al., 2017; Roy et al., 2017). Xingguang Luo’s group at the Yale University School of Medicine reported that 9,453 out of 23,677 piRNAs were detected in the human prefrontal cortex, many of which were specifically found in the prefrontal cortex compared to stomach tissues (Qiu et al., 2017; Mao et al., 2019). These 9,453 piRNAs are located in 500 piRNA clusters (Mao et al., 2019). In another study, Roy et al. identified 564 piRNAs that are mostly given rise from 148 piRNA clusters in the human brain (Roy et al., 2017). Interestingly, the expression of three out of four *PIWI* genes, *HIWI/PIWIL1*, *HILI/PIWIL2*, and *HIWI2/PIWIL4*, except *PIWIL3*, have been detected in the human brain (Roy et al., 2017).

### 2.2. Neuronal differentiation

The human embryonal carcinoma cell line, Ntera2/D1 (NT2), can undergo differentiation along the neuronal lineage by *all-trans* retinoic acid (RA) (Andrews, 1984; Pleasure and Lee, 1993; Hill et al., 2012). The RNA and protein levels of PIWIL4, a PIWI subfamily member, are consistently upregulated by RA (Subhramanyam et al., 2020). PIWIL4 is predominantly localized in the nucleus, where it interacts with lysine-specific demethylase 6A (KDM6A), also known as ubiquitously transcribed tetratricopeptide repeat, X chromosome (UTX), which is a dimethyl-or trimethyl-histone H3 lysine 27 (H3K27) demethylase. Trimethylation of H3K27 (H3K27me3) is a critical transcriptionally repressive epigenetic mark (Ferrari et al., 2014; Moody et al., 2017). Upon RA treatment, PIWIL4 mediates the removal of H3K27me3 marks from the promoters of early neuronal differentiation marker genes, such as *Homeobox A1* (*HOXA1*), *Neurogenic differentiation 1* (*NEUROD1*), and *Cytochrome P450 26A1* (*CYP26A1*), and subsequently upregulates the expression of terminal neuronal marker genes, *Microtubule-associated protein 2* (*MAP2*) and *Tubulin Beta 3 Class III* (*TUBB3*) (Subhramanyam et al., 2020; Figure 1B).

During RA-induced neuronal differentiation of NT2 cells, two piRNAs, *piR-DQ582359* and *piR-DQ596268*, were upregulated (Subhramanyam et al., 2022). Furthermore, these piRNAs potentially downregulated the expression of cold-shock domain-containing RNA-binding proteins, such as *DIS3 homolog, exosome endoribonuclease and 3′–5′ exoribonuclease* (*DIS3*), *DIS3 like 3′–5′ exoribonuclease 2* (*DIS3L2*), and *Y box-binding protein* (*YB-1*), which likely suppress the expression of *MAP2* and *TUBB3* (Subhramanyam et al., 2022), suggesting that piRNAs mediate neuronal differentiation by modulating neuronal gene expression (Figure 1B).

### 2.3. Axon regeneration

Several studies have implicated neuronal piRNAs in neuronal injury and subsequent regeneration in mammals. Nearly 4,000 out of 39,727 piRNAs have been detected in the rat cerebral cortex, some of which are differentially expressed after transient focal ischemia (Dharap et al., 2011). Likewise, several neuronal piRNAs have been detected in the rat sciatic nerve axoplasm, where they are associated with Miwi/Piwil1 proteins (Phay et al., 2018). Moreover, depletion of *Miwi/Piwil1* in cultured rat peripheral neurons increased axon regrowth after axonal injury, suggesting that neuronal piRNAs negatively regulate neural repair and regenerative processes following injury (Phay et al., 2018).

In mouse Schwann cells, glial cells of the peripheral nervous system (PNS) that support neurons (Bhatheja and Field, 2006), thousands of piRNAs are differentially expressed after nerve transection (Sohn et al., 2019). In addition, although the expression level of the Miwi/Piwil1 protein was dramatically reduced after sciatic nerve injury, an upregulated piRNA, *piR009614*, enhanced the migration of Schwann cells in mice, suggesting that piRNAs are associated with neuronal responses and axon-glial contact during peripheral nerve injury (Sohn et al., 2019).

### 2.4. Learning, memory, and behavior

Several studies have shown that neuronal piRNAs are functionally involved in learning and memory in mice. *Mili/Piwil2*-knockout mice show hyperactivity and reduced anxiety-like behaviors (Nandi et al., 2016; Leighton et al., 2019). Remarkably, the simultaneous knockdown of *Miwi/Piwil1* and *Mili/Piwil2* in the dorsal hippocampus increased freezing behavior during fear conditioning, indicating an enhanced contextual fear memory. Together, these findings suggest that neuronal piRNAs potentially interfere with the performance of behavioral tasks relevant to learning and memory (Leighton et al., 2019).

Moreover, one of the highly expressed piRNAs in the mouse CNS, *piR-DQ541777* (Lee et al., 2011), was significantly upregulated in a mouse model of neuropathic pain induced by chronic constriction injury (CCI) of sciatic nerve and increased DNA methylation levels on the promoter of *CDK5 regulatory subunit-associated protein 1* (*Cdk5rap1*) by recruiting DNA methyltransferase 3A (DNMT3a) (Zhang et al., 2019). Cdk5rap1 is an endogenous inhibitor of Cdk5 that contributes to pain information processing in the spinal cord (Ching et al., 2002; Fang-Hu et al., 2015; Liu et al., 2018; Moutal et al., 2019). These observations indicated the presence of piRNA-mediated regulation of neuropathic pain.

## 3. piRNA and neurodegenerative diseases

We have reviewed the association between piRNAs and neurodegenerative diseases (Table 1).

**Table 1:** List of piRNAs associated with the neural processes and neurodegenerative diseases.

Diseases	Tissues or cells	Change in RNA expression levels	Results	References
AD	Prefrontal cortex	81 piRNAs upregulated, 22 piRNAs downregulated	Most of these piRNAs are correlated with the genome-wide significant AD risk SNPs 10 piRNAs are associated with survival	Qiu et al. (2017) and Mao et al. (2019)
AD	Brain	146 piRNAs upregulated, 3 piRNAs downregulated	4 piRNAs target 4 genes involved in the 5 most significant AD-associated pathways	Roy et al. (2017)
AD	Cerebrospinal fluid (CSF)	3 piRNAs in the CSF-derived exosomes	Potential biomarkers to distinguish AD and non-AD.	Jain et al., (2019)
AD	Cortex and cerebellum	HERV, retrotransposons, etc. upregulated	Pathogenic tau induces heterochromatin decondensation	Sun et al. (2018)
AD	Hippocampal neuron	*HIWI/PIWIL1* upregulated	Pathogenic tau induces heterochromatin decondensation	Frost et al. (2014)
PD	Prefrontal cortex	20 piRNAs differentially expressed	Different piRNA expression patterns were observed in PD and PDD, although none of the genes were targeted	Zhang and Wong (2022)
PD	Amygdala	55 piRNAs differentially expressed	Target 20 protein-coding genes and undergo dysregulation in different PD subtypes	Zhang and Wong (2022)
PD	Blood	6 piRNAs in the blood small extracellular vesicles	Potential noninvasive biomarkers for PD diagnosis	Zhang and Wong (2022)
ALS	Brain	3 piRNAs upregulated, 2 piRNAs downregulated	Differentially expressed piRNAs in the ALS brains	Abdelhamid et al. (2022)
ALS	Brain	*HIWI/PIWIL1* upregulated, *HIWI2/PIWIL4* downregulated	HIWI/PIWIL1 was co-localized with TDP-43	Abdelhamid et al. (2022)
ALS	Serum	*hsa-piR-33151*	Decreased in ALS	Waller et al. (2017)
HD	Cortical prefrontal cortex	16 piRNAs differentially expressed in HD brains	Target several genes involved in the brain physiopathology pathway	Panero et al. (2017)

### 3.1. Alzheimer’s disease (AD)

AD is the most common form of progressive dementia and is characterized by memory impairment and cognitive decline (Masters et al., 2015; Knopman et al., 2021; Sato et al., 2021). Patients with AD exhibit some pathological symptoms, including amyloid plaques, which are mainly composed of aggregates of amyloid beta (Aβ) peptides, and neurofibrillary tangles (NFTs), composed of hyperphosphorylated tau filaments that are further linked with neurodegenerative tauopathies and synaptic loss in the brain (McKhann et al., 1984; Braak and Braak, 1990, 1991, 1996; Serrano-Pozo et al., 2011; DeTure and Dickson, 2019).

In AD hippocampal neurons, the expression level of *HIWI/PIWIL1* is increased, probably due to global heterochromatin relaxation induced by pathogenic tau aggregates (Frost et al., 2014; Table 1). Consistent with this, numerous piRNAs have been shown to be upregulated in AD brains, most of which harbor complementary target gene transcripts (Qiu et al., 2017; Roy et al., 2017). Qiu et al. (2017) have demonstrated that 103 of 9,453 human brain piRNAs were differentially expressed in AD brains, of which 81 piRNAs were upregulated and the remaining were downregulated. Most of these piRNAs correlated with genome-wide significant AD risk single nucleotide polymorphisms (SNPs); e.g., the expression levels of *piR-DQ581734*, *piR-DQ592330*, and *piR-DQ600318* correlated with AD risk SNPs in the *Apolipoprotein E* (*APOE*) cluster, and *piR-DQ597397* correlated with SNPs in the *APOJ* cluster (Qiu et al., 2017). Of note, among the 9,453 brain piRNAs, 10 piRNAs were significantly associated with years of survival, suggesting a potential role of neuronal piRNAs in lifespan determination (Mao et al., 2019). Roy et al. (2017) identified 149 piRNAs that were differentially expressed in AD brains, of which the expression levels of 146 piRNAs were upregulated, while only three piRNAs were downregulated. Target gene prediction of these dysregulated piRNAs revealed the five most significant AD-associated pathways enriched with four genes, *Cytochrome C somatic* (*CYCS*) in “Apoptosis and survival_BAD phosphorylation” and “Development_PACAP signaling in neural cells,” *Karyopherin α6* (*KPNA6*) also known as *Importin α7* (*IMPα7*) in “Neurophysiological process_Dynein-dynactin motor complex in axonal transport in neurons,” *Ras-related protein Rab-11A* (*RAB11A*) in “Neurophysiological process_Constitutive and activity-dependent synaptic AMPA receptor delivery,” and *Lin-7 homolog C* (*LIN7C*) in “Neurophysiological process_Constitutive and regulated NMDA receptor trafficking,” that were regulated by 4 piRNAs; *piR-38240* was predicted to target *CYCS* and *KPNA6*, *piR-34393* targets *CYCS* and *RAB11A*, *piR-40666* targets *KPNA6*, and *piR-51810* targets *LIN7C* (Roy et al., 2017). Notably, several piRNAs were commonly identified as AD-associated piRNAs in two independent studies by Qiu et al. and Roy et al. (Huang and Wong, 2021), suggesting the presence of consistently dysregulated piRNAs in AD brains and their utility as potential biomarkers for AD.

Sun et al. (2018) showed that the transcription of specific subsets of transposons, such as human endogenous retroviruses (HERVs) and retrotransposons, is enhanced in AD brains and brains with progressive supranuclear palsy (PSP), a form of neurodegenerative tauopathy. Pathogenic tau aggregates may promote neuronal cell death through heterochromatin decondensation and aberrant expression of *PIWI* and piRNAs, caused by transposon dysregulation in AD and neurodegenerative tauopathies.

Jain et al. conducted a pioneering preclinical study regarding the identification and development of piRNAs as diagnostic biomarkers and possible therapeutic agents for AD and analyzed the piRNA expression profile in the sncRNAome in exosomes derived from human cerebrospinal fluid (CSF) (Jain et al., 2019). They demonstrated that the expression levels of *piR_019324* were decreased, whereas those of *piR_019949* and *piR_020364* were increased in the CSF-derived exosomes of patients with AD. Importantly, changes in sncRNA levels, termed sncRNA signature, consisting of the three piRNAs and three miRNAs, miR-27a-3p, miR-30a-5p, and miR-34c, which are all upregulated in the CSF of patients with AD and have been linked to memory function and neurodegeneration (Zovoilis et al., 2011; Croce et al., 2013; Sala Frigerio et al., 2013; Müller et al., 2014), could distinguish patients with AD from non-AD individuals with an area under the curve (AUC) value of 0.83 (Jain et al., 2019). Aβ40 and Aβ42 are the major forms of Aβ in the brain, and Aβ42 is much more likely to form aggregates and is more toxic to neurons than Aβ40 (Yan and Wang, 2006; Hampel et al., 2021). Therefore, measurement of the CSF Aβ42/Aβ40 ratio and phosphorylated tau (p-tau) can be used to distinguish between clinical and preclinical AD (Jack et al., 2018; Soria Lopez et al., 2019; Campbell et al., 2021). Importantly, combined measurements of the Aβ42/40 ratio and pTau predicted with an AUC of 0.59, while on combining the sncRNA signature with them, the AUC value reached 0.98 (Jain et al., 2019). Together, piRNAs could be candidate biomarkers for AD, and the CSF-derived exosomal sncRNA signature may have the potential to identify AD individuals with high sensitivity and specificity.

### 3.2. Parkinson’s disease (PD)

PD is the second most common neurodegenerative disease after AD with major neuropathological features, including the gradual loss of dopaminergic neurons in the substantia nigra pars compacta and the presence of Lewy bodies and Lewy neurites in the neurons of PD brains (Bernheimer et al., 1973; Kalia and Lang, 2015; Bloem et al., 2021).

Zhang and Wong detected 296 and 508 out of 902 somatic piRNAs in the prefrontal cortex and amygdala that is a frequent site of Lewy bodies, respectively, of which, 20 and 55 piRNAs were differentially expressed in PD (Zhang and Wong, 2022; Table 1). They further found that 55 piRNAs that were differentially expressed in the amygdala of patients with PD were predicted to target 20 protein-coding genes, including *Mitochondrially encoded cytochrome C oxidase I* (*MT-CO1*) and *MT-CO3* (Zhang and Wong, 2022), which encode the protein components of respiratory complex IV of the electron transport chain and are implicated in PD (Bartels and Leenders, 2010; Foti et al., 2019; Choong et al., 2021). In addition, changes in piRNA expression levels were also observed in different PD stages and subtypes, such as the premotor and motor stages of PD and Parkinson’s disease dementia (PDD), a PD-associated dementia (Goldman and Postuma, 2014; Weil et al., 2017), suggesting the possible involvement of piRNAs in the onset and progression of PD (Zhang and Wong, 2022).

Moreover, the expression levels of six piRNAs, *piR-has-92056*, *piR-hsa-150797*, *piR-hsa-347751*, *piR-hsa-1909905*, *piR-hsa-2476630*, and *piR-hsa-2834636* in blood small extracellular vesicles showed the highest relevance to PD, with an AUC value of 0.89 using a sparse partial least square discriminant analysis (sPLS-DA), suggesting that these piRNAs can be potential noninvasive biomarkers for PD diagnosis (Zhang and Wong, 2022).

### 3.3. Amyotrophic lateral sclerosis (ALS)

ALS is a progressive neurodegenerative disease that affects nerve cells in the brain and spinal cord (Hardiman et al., 2017; van Es et al., 2017; Zucchi et al., 2020). The vast majority of cases (about 90–95%) are sporadic and have no known cause, while the remaining 5–10% are hereditary and are termed familial ALS (Talbott et al., 2016; Prasad et al., 2019). One of the pathological hallmarks of ALS is the aberrant aggregation of the TAR DNA-binding protein 43 (TDP-43) protein in neuronal cells of the brains (Brown and Al-Chalabi, 2017; Hardiman et al., 2017; Nguyen et al., 2018). TDP-43 is an RNA-binding protein belonging to the heterogeneous nuclear ribonucleoprotein (hnRNP) family, suggesting dysregulated RNA metabolism of multiple RNAs, including piRNAs, during the disease processes of ALS (Prasad et al., 2019).

In human brains with sporadic ALS, three piRNAs, *hsa-piR-000578*, *hsa-piR-020871*, and *hsa-piR-022184*, were upregulated, whereas two piRNAs, *hsa-piR-009294* and *hsa-piR-016735*, were downregulated (Abdelhamid et al., 2022; Table 1). The expression level of *HIWI/PIWIL1* was also increased in sporadic ALS brain tissues, whereas that of *HIWI2/PIWIL4* was decreased (Abdelhamid et al., 2022). Furthermore, HIWI/PIWIL1 proteins were co-localized with TDP-43 in the motor neurons of sporadic ALS lumbar cords, suggesting that HIWI/PIWIL1 may contribute to the formation of TDP-43 inclusions (Abdelhamid et al., 2022). In addition, the expression level of the piRNA, *hsa-piR-33151*, has been reported to be decreased in the serum of patients with ALS (Waller et al., 2017). Taken together, these observations imply that dysregulation of piRNAs is linked to the pathogenesis of ALS and that piRNAs can be potential diagnostic biomarkers and therapeutic targets of ALS.

### 3.4. Huntington’s disease (HD)

HD is a mostly inherited neurodegenerative disease caused by a CAG trinucleotide repeat expansion in the *Huntingtin* (*HTT*) gene, which leads to progressive motor dysfunction, cognitive impairment, and psychiatric disturbance due to the gradual loss of neurons, predominantly in the striatum within the brain (MacDonald et al., 1993; Julien et al., 2007; Walker, 2007; Illarioshkin et al., 2018; Tabrizi et al., 2020).

Panero et al. developed an analytical pipeline, the integrative Small RNA Tool-kit (iSmaRT), for the analysis of sncRNA-seq data and reevaluated the sncRNA-seq datasets derived from HD cortical prefrontal cortex tissues (Hoss et al., 2015) with iSmaRT (Panero et al., 2017). Sixteen piRNAs were differentially expressed in HD brains. Target prediction, followed by reactome pathway enrichment analysis, revealed several target genes involved in pathways related to brain physiopathology (Table 1), *Caspase-8 (CASP8) and Fas-associated protein with death domain (FADD) like apoptosis regulator* (*CFLAR*) in “CASP8 activity” (Strand et al., 2005) and *Neural precursor cell expressed developmentally downregulated gene 4-like* (*NEDD4L*) in “Downregulation of TGF-beta receptor signaling/Downregulation of SMAD2/3:SMAD4 transcriptional activity” (Ding et al., 2013), suggesting a possible role of the brain piRNAs in HD pathogenicity.

## 4. Conclusion

Mounting evidence has shown that piRNAs are involved in various neuronal events. Furthermore, dysregulation of neuronal piRNAs and their regulatory gene networks has been implicated in the pathology of various neurodegenerative diseases including AD. We are just beginning to understand the role of neuronal piRNAs in these neuronal events and the development of neurodegenerative diseases. In most cases, the molecular mechanisms underlying neuronal piRNA-mediated gene regulation remain largely enigmatic. Furthermore, several neurodegenerative diseases have shown increased transposon expression (Sun et al., 2018), yet the involvement of neuronal piRNAs in transposon activation remains elusive. Further investigation of neuronal piRNA activity and function could uncover their exact target genes and their contribution to regulatory gene networks associated with neuronal processes and diseases. This also leads to an understanding of the precise mechanisms that help develop novel therapeutic strategies based on neuronal piRNAs that could work in the diagnosis and/or treatment of various neurodegenerative diseases.

## Author contributions

All authors listed have made a substantial, direct, and intellectual contribution to the work and approved it for publication.

## Funding

This work was supported by grants from the Japan Society for the Promotion of Science (JSPS) (no. 20K06596 to KS), (no. 20K07350 to K-iT), and (no. 21H04829 to SI), the Kowa Life Science Foundation (KS), Kanzawa Medical Research Foundation (KS), Takeda Science Foundation (K-iT and SI), Naito Foundation (K-iT), and IRIDE in TMIG (KS).

## Conflict of interest

The authors declare that the research was conducted in the absence of any commercial or financial relationships that could be construed as a potential conflict of interest.

## Publisher’s note

All claims expressed in this article are solely those of the authors and do not necessarily represent those of their affiliated organizations, or those of the publisher, the editors and the reviewers. Any product that may be evaluated in this article, or claim that may be made by its manufacturer, is not guaranteed or endorsed by the publisher.

## References

[ref1] Abdelhamid R. F. Ogawa K. Beck G. Ikenaka K. Takeuchi E. Yasumizu Y.. (2022). piRNA/PIWI protein complex as a potential biomarker in sporadic amyotrophic lateral sclerosis. Mol. Neurobiol. 59, 1693–1705. doi: 10.1007/s12035-021-02686-2, PMID: 35015250 PMC8882100

[ref2] Andrews P. W. (1984). Retinoic acid induces neuronal differentiation of a cloned human embryonal carcinoma cell line *in vitro*. Dev. Biol. 103, 285–293. doi: 10.1016/0012-1606(84)90316-6, PMID: 6144603

[ref01] Aravin A. Gaidatzis D. Pfeffer S. Lagos-Quintana M. Landgraf P. Iovino N.. (2006). A novel class of small RNAs bind to MILI protein in mouse testes. Nature 442, 203–207. doi: 10.1038/nature04916 16751777

[ref3] Bartels A. L. Leenders K. L. (2010). Cyclooxygenase and neuroinflammation in Parkinson’s disease neurodegeneration. Curr. Neuropharmacol. 8, 62–68. doi: 10.2174/157015910790909485, PMID: 20808546 PMC2866462

[ref4] Bernheimer H. Birkmayer W. Hornykiewicz O. Jellinger K. Seitelberger F. (1973). Brain dopamine and the syndromes of Parkinson and Huntington. Clinical, morphological and neurochemical correlations. J. Neurol. Sci. 20, 415–455. doi: 10.1016/0022-510x(73)90175-5, PMID: 4272516

[ref5] Bhatheja K. Field J. (2006). Schwann cells: origins and role in axonal maintenance and regeneration. Int. J. Biochem. Cell Biol. 38, 1995–1999. doi: 10.1016/j.biocel.2006.05.007, PMID: 16807057

[ref6] Bloem B. R. Okun M. S. Klein C. (2021). Parkinson’s disease. Lancet 397, 2284–2303. doi: 10.1016/S0140-6736(21)00218-X 33848468

[ref7] Braak H. Braak E. (1990). Alzheimer’s disease: striatal amyloid deposits and neurofibrillary changes. J. Neuropathol. Exp. Neurol. 49, 215–224. doi: 10.1097/00005072-199005000-00003 1692337

[ref8] Braak H. Braak E. (1991). Neuropathological stageing of Alzheimer-related changes. Acta Neuropathol. 82, 239–259. doi: 10.1007/BF00308809, PMID: 1759558

[ref9] Braak H. Braak E. (1996). Development of Alzheimer-related neurofibrillary changes in the neocortex inversely recapitulates cortical myelogenesis. Acta Neuropathol. 92, 197–201. doi: 10.1007/s004010050508, PMID: 8841666

[ref10] Brennecke J. Aravin A. A. Stark A. Dus M. Kellis M. Sachidanandam R.. (2007). Discrete small RNA-generating loci as master regulators of transposon activity in drosophila. Cells 128, 1089–1103. doi: 10.1016/j.cell.2007.01.043, PMID: 17346786

[ref11] Brown R. H. Al-Chalabi A. (2017). Amyotrophic lateral sclerosis. N. Engl. J. Med. 377, 162–172. doi: 10.1056/NEJMra1603471 28700839

[ref12] Campbell M. R. Ashrafzadeh-Kian S. Petersen R. C. Mielke M. M. Syrjanen J. A. van Harten A. C.. (2021). P-tau/Aβ42 and Aβ42/40 ratios in CSF are equally predictive of amyloid PET status. Alzheimers Dement. 13:e12190. doi: 10.1002/dad2.12190, PMID: 34027020 PMC8129859

[ref13] Ching Y. P. Pang A. S. Lam W. H. Qi R. Z. Wang J. H. (2002). Identification of a neuronal Cdk5 activator-binding protein as Cdk5 inhibitor. J. Biol. Chem. 277, 15237–15240. doi: 10.1074/jbc.C200032200, PMID: 11882646

[ref14] Choong C. J. Okuno T. Ikenaka K. Baba K. Hayakawa H. Koike M.. (2021). Alternative mitochondrial quality control mediated by extracellular release. Autophagy 17, 2962–2974. doi: 10.1080/15548627.2020.1848130, PMID: 33218272 PMC8525996

[ref15] Croce N. Gelfo F. Ciotti M. T. Federici G. Caltagirone C. Bernardini S.. (2013). NPY modulates miR-30a-5p and BDNF in opposite direction in an *in vitro* model of Alzheimer disease: a possible role in neuroprotection. Mol. Cell. Biochem. 376, 189–195. doi: 10.1007/s11010-013-1567-0, PMID: 23358924

[ref16] Czech B. Munafò M. Ciabrelli F. Eastwood E. L. Fabry M. H. Kneuss E.. (2018). piRNA-guided genome defense: from biogenesis to silencing. Annu. Rev. Genet. 52, 131–157. doi: 10.1146/annurev-genet-120417-031441, PMID: 30476449 PMC10784713

[ref17] DeTure M. A. Dickson D. W. (2019). The neuropathological diagnosis of Alzheimer’s disease. Mol. Neurodegener. 14:32. doi: 10.1186/s13024-019-0333-5, PMID: 31375134 PMC6679484

[ref18] Dharap A. Nakka V. P. Vemuganti R. (2011). Altered expression of PIWI RNA in the rat brain after transient focal ischemia. Stroke 42, 1105–1109. doi: 10.1161/STROKEAHA.110.598391, PMID: 21311060 PMC3566794

[ref19] Ding Y. Zhang Y. Xu C. Tao Q. H. Chen Y. G. (2013). HECT domain-containing E3 ubiquitin ligase NEDD4L negatively regulates Wnt signaling by targeting dishevelled for proteasomal degradation. J. Biol. Chem. 288, 8289–8298. doi: 10.1074/jbc.M112.433185, PMID: 23396981 PMC3605647

[ref20] Fang-Hu Zhang H. H. Yang B. X. Huang J. L. Shun J. L. Kong F. J.. (2015). Cdk5 contributes to inflammation-induced thermal hyperalgesia mediated by the p38 MAPK pathway in microglia. Brain Res. 1619, 166–175. doi: 10.1016/j.brainres.2015.01.056 25819553

[ref21] Ferrari K. J. Scelfo A. Jammula S. Cuomo A. Barozzi I. Stützer A.. (2014). Polycomb-dependent H3K27me1 and H3K27me2 regulate active transcription and enhancer fidelity. Mol. Cell 53, 49–62. doi: 10.1016/j.molcel.2013.10.030, PMID: 24289921

[ref22] Foti S. C. Hargreaves I. Carrington S. Kiely A. P. Houlden H. Holton J. L. (2019). Cerebral mitochondrial electron transport chain dysfunction in multiple system atrophy and Parkinson’s disease. Sci. Rep. 9:6559. doi: 10.1038/s41598-019-42902-7, PMID: 31024027 PMC6484105

[ref23] Frost B. Hemberg M. Lewis J. Feany M. B. (2014). Tau promotes neurodegeneration through global chromatin relaxation. Nat. Neurosci. 17, 357–366. doi: 10.1038/nn.3639, PMID: 24464041 PMC4012297

[ref24] Ghildiyal M. Seitz H. Horwich M. D. Li C. Du T. Lee S.. (2008). Endogenous siRNAs derived from transposons and mRNAs in drosophila somatic cells. Science 320, 1077–1081. doi: 10.1126/science.1157396 18403677 PMC2953241

[ref25] Girard A. Sachidanandam R. Hannon G. J. Carmell M. A. (2006). A germline-specific class of small RNAs binds mammalian Piwi proteins. Nature 442, 199–202. doi: 10.1038/nature04917, PMID: 16751776

[ref26] Goldman J. G. Postuma R. (2014). Premotor and nonmotor features of Parkinson’s disease. Curr. Opin. Neurol. 27, 434–441. doi: 10.1097/WCO.0000000000000112, PMID: 24978368 PMC4181670

[ref27] Goriaux C. Desset S. Renaud Y. Vaury C. Brasset E. (2014). Transcriptional properties and splicing of the flamenco piRNA cluster. EMBO Rep. 15, 411–418. doi: 10.1002/embr.201337898, PMID: 24562610 PMC3989672

[ref28] Hampel H. Hardy J. Blennow K. Chen C. Perry G. Kim S. H.. (2021). The amyloid-β pathway in Alzheimer’s disease. Mol. Psychiatry 26, 5481–5503. doi: 10.1038/s41380-021-01249-0, PMID: 34456336 PMC8758495

[ref29] Hardiman O. Al-Chalabi A. Chio A. Corr E. M. Logroscino G. Robberecht W.. (2017). Amyotrophic lateral sclerosis. Nat. Rev. Dis. Primers. 3:17071. doi: 10.1038/nrdp.2017.71 28980624

[ref30] Hill E. J. Jiménez-González C. Tarczyluk M. Nagel D. A. Coleman M. D. Parri H. R. (2012). NT2 derived neuronal and astrocytic network signalling. PLoS One 7:e36098. doi: 10.1371/journal.pone.0036098, PMID: 22567128 PMC3342170

[ref31] Hoss A. G. Labadorf A. Latourelle J. C. Kartha V. K. Hadzi T. C. Gusella J. F.. (2015). miR-10b-5p expression in Huntington’s disease brain relates to age of onset and the extent of striatal involvement. BMC Med. Genet. 8:10. doi: 10.1186/s12920-015-0083-3, PMID: 25889241 PMC4349621

[ref32] Huang X. Wong G. (2021). An old weapon with a new function: PIWI-interacting RNAs in neurodegenerative diseases. Transl. Neurodegener. 10:9. doi: 10.1186/s40035-021-00233-6, PMID: 33685517 PMC7938595

[ref33] Illarioshkin S. N. Klyushnikov S. A. Vigont V. A. Seliverstov Y. A. Kaznacheyeva E. V. (2018). Molecular pathogenesis in Huntington’s disease. Biochemistry 83, 1030–1039. doi: 10.1134/S0006297918090043 30472941

[ref34] Jack C. R. Bennett D. A. Blennow K. Carrillo M. C. Dunn B. Haeberlein S. B.. (2018). NIA-AA research framework: toward a biological definition of Alzheimer’s disease. Alzheimers Dement. 14, 535–562. doi: 10.1016/j.jalz.2018.02.018, PMID: 29653606 PMC5958625

[ref35] Jain G. Stuendl A. Rao P. Berulava T. Pena Centeno T. Kaurani L.. (2019). A combined miRNA-piRNA signature to detect Alzheimer’s disease. Transl. Psychiatry 9:250. doi: 10.1038/s41398-019-0579-2, PMID: 31591382 PMC6779890

[ref36] Julien C. L. Thompson J. C. Wild S. Yardumian P. Snowden J. S. Turner G.. (2007). Psychiatric disorders in preclinical Huntington’s disease. J. Neurol. Neurosurg. Psychiatry 78, 939–943. doi: 10.1136/jnnp.2006.103309, PMID: 17178819 PMC2117854

[ref37] Kalia L. V. Lang A. E. (2015). Parkinson’s disease. Lancet 386, 896–912. doi: 10.1016/S0140-6736(14)61393-3 25904081

[ref38] Kim K. W. (2019). PIWI proteins and piRNAs in the nervous system. Mol. Cells 42, 828–835. doi: 10.14348/molcells.2019.0241, PMID: 31838836 PMC6939654

[ref39] Kim V. N. Han J. Siomi M. C. (2009). Biogenesis of small RNAs in animals. Nat. Rev. Mol. Cell Biol. 10, 126–139. doi: 10.1038/nrm2632 19165215

[ref40] Knopman D. S. Amieva H. Petersen R. C. Chételat G. Holtzman D. M. Hyman B. T.. (2021). Alzheimer disease. Nat. Rev. Dis. Primers. 7:33. doi: 10.1038/s41572-021-00269-y, PMID: 33986301 PMC8574196

[ref41] Kozomara A. Birgaoanu M. Griffiths-Jones S. (2019). miRBase: from microRNA sequences to function. Nucleic Acids Res. 47, D155–D162. doi: 10.1093/nar/gky1141, PMID: 30423142 PMC6323917

[ref42] Ku H. Y. Lin H. (2014). PIWI proteins and their interactors in piRNA biogenesis, germline development and gene expression. Natl. Sci. Rev. 1, 205–218. doi: 10.1093/nsr/nwu014, PMID: 25512877 PMC4265212

[ref43] Lauretti E. Dabrowski K. Praticò D. (2021). The neurobiology of non-coding RNAs and Alzheimer’s disease pathogenesis: pathways, mechanisms and translational opportunities. Ageing Res. Rev. 71:101425. doi: 10.1016/j.arr.2021.101425, PMID: 34384901

[ref44] Lee E. J. Banerjee S. Zhou H. Jammalamadaka A. Arcila M. Manjunath B. S.. (2011). Identification of piRNAs in the central nervous system. RNA 17, 1090–1099. doi: 10.1261/rna.2565011, PMID: 21515829 PMC3096041

[ref45] Leighton L. J. Wei W. Marshall P. R. Ratnu V. S. Li X. Zajaczkowski E. L.. (2019). Disrupting the hippocampal Piwi pathway enhances contextual fear memory in mice. Neurobiol. Learn. Mem. 161, 202–209. doi: 10.1016/j.nlm.2019.04.002, PMID: 30965112

[ref46] Liu K. C. Leuckx G. Sakano D. Seymour P. A. Mattsson C. L. Rautio L.. (2018). Inhibition of Cdk5 promotes β-cell differentiation from ductal progenitors. Diabetes 67, 58–70. doi: 10.2337/db16-1587, PMID: 28986398 PMC6463766

[ref47] MacDonald M. E. Ambrose C. M. Duyao M. P. Myers R. H. Lin C. Srinidhi L.. (1993). A novel gene containing a trinucleotide repeat that is expanded and unstable on Huntington’s disease chromosomes. Cells 72, 971–983. doi: 10.1016/0092-8674(93)90585-e 8458085

[ref48] Mao Q. Fan L. Wang X. Lin X. Cao Y. Zheng C.. (2019). Transcriptome-wide piRNA profiling in human brains for aging genetic factors. Jacobs J. Genet. 4:14. PMC7059831 32149191

[ref49] Masters C. L. Bateman R. Blennow K. Rowe C. C. Sperling R. A. Cummings J. L. (2015). Alzheimer’s disease. Nat. Rev. Dis. Primers. 1:15056. doi: 10.1038/nrdp.2015.56 27188934

[ref50] McKhann G. Drachman D. Folstein M. Katzman R. Price D. Stadlan E. M. (1984). Clinical diagnosis of Alzheimer’s disease: report of the NINCDS-ADRDA work group under the auspices of Department of Health and Human Services Task Force on Alzheimer’s disease. Neurology 34, 939–944. doi: 10.1212/wnl.34.7.939, PMID: 6610841

[ref51] Moody J. D. Levy S. Mathieu J. Xing Y. Kim W. Dong C.. (2017). First critical repressive H3K27me3 marks in embryonic stem cells identified using designed protein inhibitor. Proc. Natl. Acad. Sci. U. S. A. 114, 10125–10130. doi: 10.1073/pnas.1706907114, PMID: 28864533 PMC5617284

[ref52] Moutal A. Luo S. Largent-Milnes T. M. Vanderah T. W. Khanna R. (2019). Cdk5-mediated CRMP2 phosphorylation is necessary and sufficient for peripheral neuropathic pain. Neurobiol. Pain 5:100022. doi: 10.1016/j.ynpai.2018.07.003, PMID: 31080913 PMC6505708

[ref53] Müller M. Kuiperij H. B. Claassen J. A. Küsters B. Verbeek M. M. (2014). MicroRNAs in Alzheimer’s disease: differential expression in hippocampus and cell-free cerebrospinal fluid. Neurobiol. Aging 35, 152–158. doi: 10.1016/j.neurobiolaging.2013.07.005 23962497

[ref02] Nandi S. Chandramohan D. Fioriti L. Melnick A. M. Hébert J. M. Mason C. E.. (2016). Roles for small noncoding RNAs in silencing of retrotransposons in the mammalian brain. Proc. Natl. Acad. Sci. USA 113, 12697–12702. doi: 10.1073/pnas.1609287113 27791114 PMC5111663

[ref54] Nguyen H. P. Van Broeckhoven C. van der Zee J. (2018). ALS genes in the genomic era and their implications for FTD. Trends Genet. 34, 404–423. doi: 10.1016/j.tig.2018.03.001, PMID: 29605155

[ref55] Panero R. Rinaldi A. Memoli D. Nassa G. Ravo M. Rizzo F.. (2017). iSmaRT: a toolkit for a comprehensive analysis of small RNA-Seq data. Bioinformatics 33, 938–940. doi: 10.1093/bioinformatics/btw734, PMID: 28057684

[ref56] Parhad S. S. Theurkauf W. E. (2019). Rapid evolution and conserved function of the piRNA pathway. Open Biol. 9:180181. doi: 10.1098/rsob.180181, PMID: 30958115 PMC6367137

[ref57] Perera B. P. U. Tsai Z. T. Colwell M. L. Jones T. R. Goodrich J. M. Wang K.. (2019). Somatic expression of piRNA and associated machinery in the mouse identifies short, tissue-specific piRNA. Epigenetics 14, 504–521. doi: 10.1080/15592294.2019.1600389, PMID: 30955436 PMC6557559

[ref58] Phay M. Kim H. H. Yoo S. (2018). Analysis of piRNA-like small non-coding RNAs present in axons of adult sensory neurons. Mol. Neurobiol. 55, 483–494. doi: 10.1007/s12035-016-0340-2, PMID: 27966078 PMC5901696

[ref59] Pleasure S. J. Lee V. M. (1993). NTera 2 cells: a human cell line which displays characteristics expected of a human committed neuronal progenitor cell. J. Neurosci. Res. 35, 585–602. doi: 10.1002/jnr.490350603 8411264

[ref60] Prasad A. Bharathi V. Sivalingam V. Girdhar A. Patel B. K. (2019). Molecular mechanisms of TDP-43 Misfolding and pathology in amyotrophic lateral sclerosis. Front. Mol. Neurosci. 12:25. doi: 10.3389/fnmol.2019.00025, PMID: 30837838 PMC6382748

[ref61] Qiu W. Guo X. Lin X. Yang Q. Zhang W. Zhang Y.. (2017). Transcriptome-wide piRNA profiling in human brains of Alzheimer’s disease. Neurobiol. Aging 57, 170–177. doi: 10.1016/j.neurobiolaging.2017.05.020, PMID: 28654860 PMC5542056

[ref62] Rajasethupathy P. Antonov I. Sheridan R. Frey S. Sander C. Tuschl T.. (2012). A role for neuronal piRNAs in the epigenetic control of memory-related synaptic plasticity. Cells 149, 693–707. doi: 10.1016/j.cell.2012.02.057, PMID: 22541438 PMC3442366

[ref63] Rojas-Ríos P. Simonelig M. (2018). piRNAs and PIWI proteins: regulators of gene expression in development and stem cells. Development 145:dev161786. doi: 10.1242/dev.161786 30194260

[ref64] Roy J. Sarkar A. Parida S. Ghosh Z. Mallick B. (2017). Small RNA sequencing revealed dysregulated piRNAs in Alzheimer’s disease and their probable role in pathogenesis. Mol. Biosyst. 13, 565–576. doi: 10.1039/c6mb00699j, PMID: 28127595

[ref65] Sala Frigerio C. Lau P. Salta E. Tournoy J. Bossers K. Vandenberghe R.. (2013). Reduced expression of hsa-miR-27a-3p in CSF of patients with Alzheimer disease. Neurology 81, 2103–2106. doi: 10.1212/01.wnl.0000437306.37850.22, PMID: 24212398

[ref66] Sasaki T. Shiohama A. Minoshima S. Shimizu N. (2003). Identification of eight members of the Argonaute family in the human genome. Genomics 82, 323–330. doi: 10.1016/s0888-7543(03)00129-0, PMID: 12906857

[ref67] Sato K. Siomi M. C. (2018). Two distinct transcriptional controls triggered by nuclear Piwi-piRISCs in the drosophila piRNA pathway. Curr. Opin. Struct. Biol. 53, 69–76. doi: 10.1016/j.sbi.2018.06.005, PMID: 29990672

[ref68] Sato K. Siomi M. C. (2020). The piRNA pathway in drosophila ovarian germ and somatic cells. Proc. Jpn. Acad. Ser. B Phys. Biol. Sci. 96, 32–42. doi: 10.2183/pjab.96.003, PMID: 31932527 PMC6974405

[ref69] Sato K. Takayama K. I. Hashimoto M. Inoue S. (2021). Transcriptional and post-transcriptional regulations of amyloid-β precursor protein (APP) mRNA. Front. Aging 2:721579. doi: 10.3389/fragi.2021.721579, PMID: 35822056 PMC9261399

[ref70] Serrano-Pozo A. Frosch M. P. Masliah E. Hyman B. T. (2011). Neuropathological alterations in Alzheimer disease. Cold Spring Harb. Perspect. Med. 1:a006189. doi: 10.1101/cshperspect.a006189, PMID: 22229116 PMC3234452

[ref71] Siomi M. C. Sato K. Pezic D. Aravin A. A. (2011). PIWI-interacting small RNAs: the vanguard of genome defence. Nat. Rev. Mol. Cell Biol. 12, 246–258. doi: 10.1038/nrm3089, PMID: 21427766

[ref72] Sohn E. J. Jo Y. R. Park H. T. (2019). Downregulation MIWI-piRNA regulates the migration of Schwann cells in peripheral nerve injury. Biochem. Biophys. Res. Commun. 519, 605–612. doi: 10.1016/j.bbrc.2019.09.008, PMID: 31540693

[ref73] Soria Lopez J. A. González H. M. Léger G. C. (2019). Alzheimer’s disease. Handb. Clin. Neurol. 167, 231–255. doi: 10.1016/B978-0-12-804766-8.00013-3 31753135

[ref74] Strand A. D. Aragaki A. K. Shaw D. Bird T. Holton J. Turner C.. (2005). Gene expression in Huntington’s disease skeletal muscle: a potential biomarker. Hum. Mol. Genet. 14, 1863–1876. doi: 10.1093/hmg/ddi192, PMID: 15888475

[ref75] Subhramanyam C. S. Cao Q. Wang C. Heng Z. S. L. Zhou Z. Hu Q. (2020). Role of PIWI-like 4 in modulating neuronal differentiation from human embryonal carcinoma cells. RNA Biol. 17, 1613–1624. doi: 10.1080/15476286.2020.1757896, PMID: 32372724 PMC7567516

[ref76] Subhramanyam C. S. Cao Q. Wang C. Heng Z. S. Zhou Z. Hu Q. (2022). piRNAs interact with cold-shock domain-containing RNA binding proteins and regulate neuronal gene expression during differentiation. Mol. Neurobiol. 59, 1285–1300. doi: 10.1007/s12035-021-02678-2, PMID: 34982407

[ref77] Sun W. Samimi H. Gamez M. Zare H. Frost B. (2018). Pathogenic tau-induced piRNA depletion promotes neuronal death through transposable element dysregulation in neurodegenerative tauopathies. Nat. Neurosci. 21, 1038–1048. doi: 10.1038/s41593-018-0194-1, PMID: 30038280 PMC6095477

[ref78] Tabrizi S. J. Flower M. D. Ross C. A. Wild E. J. (2020). Huntington disease: new insights into molecular pathogenesis and therapeutic opportunities. Nat. Rev. Neurol. 16, 529–546. doi: 10.1038/s41582-020-0389-4, PMID: 32796930

[ref79] Talbott E. O. Malek A. M. Lacomis D. (2016). The epidemiology of amyotrophic lateral sclerosis. Handb. Clin. Neurol. 138, 225–238. doi: 10.1016/B978-0-12-802973-2.00013-6 27637961

[ref80] Vagin V. V. Sigova A. Li C. Seitz H. Gvozdev V. Zamore P. D. (2006). A distinct small RNA pathway silences selfish genetic elements in the germline. Science 313, 320–324. doi: 10.1126/science.1129333, PMID: 16809489

[ref81] van Es M. A. Hardiman O. Chio A. Al-Chalabi A. Pasterkamp R. J. Veldink J. H.. (2017). Amyotrophic lateral sclerosis. Lancet 390, 2084–2098. doi: 10.1016/S0140-6736(17)31287-4 28552366

[ref82] Wakisaka K. T. Tanaka R. Hirashima T. Muraoka Y. Azuma Y. Yoshida H.. (2019). Novel roles of drosophila FUS and Aub responsible for piRNA biogenesis in neuronal disorders. Brain Res. 1708, 207–219. doi: 10.1016/j.brainres.2018.12.028, PMID: 30578769

[ref83] Walker F. O. (2007). Huntington’s disease. Lancet 369, 218–228. doi: 10.1016/S0140-6736(07)60111-1 17240289

[ref84] Waller R. Wyles M. Heath P. R. Kazoka M. Wollff H. Shaw P. J.. (2017). Small RNA sequencing of sporadic amyotrophic lateral sclerosis cerebrospinal fluid reveals differentially expressed miRNAs related to neural and glial activity. Front. Neurosci. 11:731. doi: 10.3389/fnins.2017.00731, PMID: 29375285 PMC5767269

[ref85] Wang C. Lin H. (2021). Roles of piRNAs in transposon and pseudogene regulation of germline mRNAs and lncRNAs. Genome Biol. 22:27. doi: 10.1186/s13059-020-02221-x, PMID: 33419460 PMC7792047

[ref86] Wang J. Shi Y. Zhou H. Zhang P. Song T. Ying Z.. (2022). piRBase: integrating piRNA annotation in all aspects. Nucleic Acids Res. 50, D265–D272. doi: 10.1093/nar/gkab1012, PMID: 34871445 PMC8728152

[ref87] Wang X. Ramat A. Simonelig M. Liu M. F. (2022). Emerging roles and functional mechanisms of PIWI-interacting RNAs. Nat. Rev. Mol. Cell Biol. 24, 123–141. doi: 10.1038/s41580-022-00528-0, PMID: 36104626

[ref88] Weil R. S. Lashley T. L. Bras J. Schrag A. E. Schott J. M. (2017). Current concepts and controversies in the pathogenesis of Parkinson’s disease dementia and dementia with Lewy bodies. F1000Res 6:1604. doi: 10.12688/f1000research.11725.1, PMID: 28928962 PMC5580419

[ref89] Wu P. H. Zamore P. D. (2021). Defining the functions of PIWI-interacting RNAs. Nat. Rev. Mol. Cell Biol. 22, 239–240. doi: 10.1038/s41580-021-00336-y, PMID: 33483697

[ref90] Yan Y. Wang C. (2006). Abeta42 is more rigid than Abeta40 at the C terminus: implications for Abeta aggregation and toxicity. J. Mol. Biol. 364, 853–862. doi: 10.1016/j.jmb.2006.09.046, PMID: 17046788

[ref91] Zhang C. Sha H. Peng Y. Wang Y. Liu C. Zhou X. (2019). PiRNA-DQ541777 contributes to neuropathic pain via targeting Cdk5rap1. J. Neurosci. 39, 9028–9039. doi: 10.1523/JNEUROSCI.1602-19.2019, PMID: 31519819 PMC6832687

[ref92] Zhang P. Si X. Skogerbø G. Wang J. Cui D. Li Y.. (2014). piRBase: A web resource assisting piRNA functional study. Database 2014, 2014:bau110. doi: 10.1093/database/bau110, PMID: 25425034 PMC4243270

[ref93] Zhang T. Wong G. (2022). Dysregulation of human somatic piRNA expression in Parkinson’s disease subtypes and stages. Int. J. Mol. Sci. 23:2469. doi: 10.3390/ijms23052469, PMID: 35269612 PMC8910154

[ref94] Zovoilis A. Agbemenyah H. Y. Agis-Balboa R. C. Stilling R. M. Edbauer D. Rao P.. (2011). microRNA-34c is a novel target to treat dementias. EMBO J. 30, 4299–4308. doi: 10.1038/emboj.2011.327, PMID: 21946562 PMC3199394

[ref95] Zucchi E. Bonetto V. Sorarù G. Martinelli I. Parchi P. Liguori R.. (2020). Neurofilaments in motor neuron disorders: towards promising diagnostic and prognostic biomarkers. Mol. Neurodegener. 15:58. doi: 10.1186/s13024-020-00406-3, PMID: 33059698 PMC7559190

